# NUCB-2/Nesfatin-1 promotes the proliferation of nasopharyngeal carcinoma cells

**DOI:** 10.1186/s12935-023-03038-x

**Published:** 2023-08-27

**Authors:** Yunlai Liang, Yating Ma, Kun Wang, Manglin Xiang, Bin Yi

**Affiliations:** Department of Clinical Laboratory, Xiangya Hospital of Central South University, No. 87, Xiangya Street, Kaifu District, Changsha, 410008 Hunan China

**Keywords:** Nasopharyngeal carcinoma, Nucleobindin 2, Nesfatin-1, Biomarker, NPC diagnosis

## Abstract

**Purpose:**

The association of NUCB-2/Nesfatin-1 with nasopharyngeal carcinoma (NPC) remains unclear. We clarified the role of NUCB-2/Nesfatin-1 in the development, progression and diagnosis of NPC.

**Materials and methods:**

In nasopharyngeal carcinoma cell lines (5-8 F, 6-10B, CNE1, CNE2 and NP69), western blotting, MTT, EdU and other techniques were performed to investigate the role of NUCB-2 in nasopharyngeal carcinoma. 70 tissue samples (39 NPC and 31 rhinitis) and 140 serum samples (including NPC, rhinitis, other head and neck tumors and healthy control) were included to explore the expression of NUCB-2 and its metabolite Nesfatin-1 in tissues or serum of patients with nasopharyngeal carcinoma.

**Results:**

NUCB-2 level in NPC tissue was higher than that in rhinitis tissue (P < 0.05). Suppression of NUCB-2 in the NPC cell line CNE2 inhibited proliferation and clone formation of the cells; on the contrary, improvement of NUCB-2 in the NPC cell line CNE1 promoted cell propagation and clone development. An elevated serum level of NUCB-2 in NPC patients was detected, compared to that in patients with other head and neck tumors, rhinitis or healthy donors. Determination of nesfatin-1 combined with EA-IgA, VCA-IgA and Rta-IgG in serum samples for NPC diagnosis reached a sensitivity of 93.6% and a specificity of 94.5%, while the positive and negative predictive value of this diagnostic model was 89.8% and 96.6%, and the accuracy yielded 94.2%.

**Conclusion:**

This study revealed that NUCB-2 could enhance proliferation of NPC cells and NUCB-2/nesfatin-1 has the potential to be a serological marker to aid early diagnosis of nasopharyngeal carcinoma.

## Introduction

Nasopharyngeal carcinoma (NPC) is an epithelial carcinoma arising from the nasopharyngeal mucosal lining. NPC is often observed at the pharyngeal recess posteromedial to the medial crura of the eustachian tube opening in the nasopharynx. According to the International Agency for Research on Cancer, in 2018, there were about 129 000 new cases of NPC, accounting for only 0.7% of all cancers diagnosed in that year, nevertheless, its geographical global distribution was extremely unbalanced as > 70% of the new cases were in the east and southeast Asia, with an age-standardized rate of 3.0 per 100 000 in China to 0.4 per 100 000 in populations that were mainly white [1, 2]. Unfortunately, 70% of the NPC patients were found locally advanced at the first diagnosis [3], leading to a poor prognosis. Therefore, it is of great significance to explore the molecular mechanism of NPC occurrence and molecular markers that can improve the early diagnosis of the patients.

Based on iTRAQ binding mass spectrometry platform, our previous study already screened and identified 208 differential proteins related to NPC occurrence and metastasis in high metastasis NPC cell line 5-8 F and low metastasis NPC cell line 6-10B, compared to immortalized normal nasopharynx epithelial cell line NP69 [4]. Of them, nucleobindin-2 (NUCB-2) was up-regulated with a 6-10B/NP69 ratio of 2.19 and a 5-8 F/NP69 ratio of 2.26, and nine repetitive peptide segments connected to the protein were detected; however, its ratio of 6-10B to 5-8 F only achieved 1.19, indicating a clear exploring value for NUCB-2 in tumorigenesis of NPC.

NUCB-2 has a molecular weight of about 40 KD and contains a variety of functional domains, including a signaling peptide, a Leu/Ile rich region, two Ca^2+^ binding domains and a leucine zipper structure [5]. NUCB-2 protein can be cut into three fragments by prohormone convertase (PC) in vivo, which are nesfatin-1 (aa 1–86), nesfatin-2 (aa 85–163) and nesfatin-3 (aa 166–396) [6]. Studies have shown that NUCB-2 and its metabolites are related to insulin resistance [7], emotion adjustment [8] and temperature regulation [9]. In recent years, NUCB-2 and its cleavage products have been found to have links with the development and metastasis of various tumors. For example, NUCB-2 expression increased in renal clear cell carcinoma [10], breast cancer [11] and prostate cancer [12]. Cytological experiments showed that NUCB-2/nesfatin-1 could inhibit the proliferation of human ovarian epithelial cancer cell through the mTOR signaling pathway and induce cell apoptosis through the RhoA/ROCK pathway [13]. Kiyoshi Takagl et al. proved that NUCB-2/nesfatin-1 was associated with the development, progression and metastasis of endometrial cancer [14]. To the best of our knowledge, there have been few published studies involved in the function of NUCB-2/nesfatin-1 in NPC. In order to address this issue, we examined the expression levels of NUCB-2 both in serum and tumor tissue from NPC patients. Furthermore, we investigated whether NUCB-2/nesfatin-1 could affect the tumor development in NPC cell line and serve as a potential biomarker for NPC diagnosis and treatment.

## Materials and methods

### Cell lines, tissues and serum samples

Cell lines of high differentiation NPC CNE1 and low differentiation NPC CNE2 were maintained in RPMI-1640 medium supplemented with 10% FBS in a humidified chamber with 5% CO2 at 37 °C. Between Jan. 2017 and Dec. 2019, in the First Xiangya Hospital of Central South University, surgically excised 39 pathological tissues from adult patients initially diagnosed with NPC and 31 rhinitis samples were collected and stored at − 80 °C. Additionally, 10 mL of venous blood from each patients with NPC(n = 49), head and neck tumor (n = 35) and rhinitis (n = 20) were collected and the sera were isolated, then aliquoted and kept at -80 °C; meanwhile, sera from 36 healthy donors were prepared.

### shRNA construction and stable clone selection

NUCB-2-targetting oligonucleotides were designed and generated from full-length human NUCB-2 gene by GeneChem (Shanghai, China). After testing the knockdown efficiencies, stem-loop DNA oligonucleotides (sense, 5-CCGGGCATGAAAATCACCCTAAACTCGAGTTTAGGGTGATTTTCATGCTTTTTG-3) were synthesized and cloned into the lentivirus-based shRNA vector GV493 by GeneChem. A non-targeting stem-loop DNA GV493 vector was also produced to serve as the negative control. Lentiviral particles were prepared as described in the reference. NUCB-2-shRNA-lentivirus was transfected into the target CNE2 cells by adding Lipofectamine™ 2000 (Invitrogen, CA, USA) under the guidance of the manufacturer’s protocol. Virus with empty vector-transfected cells were conducted as the negative control. After that, the transfected cells were maintained in medium containing 1 µg/mL puromycin to obtain stably transfected CNE2 cells with NUCB-2 knockdown, namely, CNE2-KD, as well as the negative control, CNE2-NC. Meanwhile, cells without virus transfection were used as the blank control.

To generate NPC cell line with NUCB-2 overexpression, a recombined expression vector, pLVX-Puro-NUCB-2, was established by Vigene Bioscience (Shandong, China). Briefly, the NUCB-2 fragments were synthesized by PCR amplification with the upstream primer: 5’-GATCGCTAGCGCTACCGGACTCAGATCTCGAGATGAGGTGGAGGACCATCCTGCTACAGT-3’ and downstream primer: 5’-GCGCCTCCCCTACCCGGTAGAATTATCTAGATTAAATGTGTGGCTCAAACTTCAAT-3’. After amplification, the PCR products were ligated into the pLVX-Puro vector (Vigene Bioscience), containing Xho-I and Xba-I cleavages, to construct the recombinant vector pLVX-Puro-NUCB-2, followed by transformation into *E. coli* Top10 bacteria. Then, the pLVX-Puro-NUCB-2 or the empty vectors were added into the target CNE1 cells with Lipofectamine™ 2000 for transfection and maintained in medium containing 0.5 µg/mL puromycin to screen the stably transfected CNE1 cells, which were designated as CNE1-OE, or the negative control, CNE1-NC. Untreated CNE1 cells served as the blank cobtrol.

### Western blotting

Different cell lines (CNE1, CNE2, 5-8 F, 6-10B, NP69 and Hela) were cultured in the corresponding medium and then harvested when the cell fusion rate is greater than 80%, followed by lysed in the RIPA buffer for Western blot detection. Initially, 35 µg of proteins in each sample were mixed with the Laemmli buffer (TIANGEN Biotech, Beijing, China) and subjected to sodium dodecyl sulfate-polyacrylamide gel electrophoresis (SDS-PAGE) for protein separation; following blotting onto a polyvinylidene difluoride (PVDF) membrane (Millipore, MA, USA), the blots were incubated in the 1:330 diluted primary antibody of anti-NUCB-2 (Santa Cruz, TX, USA) overnight at 4℃; after that, the goat anti-mouse antibody (Abcam, MA, USA) was added at 1:5000 dilution and kept for 1 h at room temperature. The signal was visualized with Luminata Crescendo Western HRP Substrate (Millipore, MA, USA) and quantitated by Storm Optical Scanner, a densitometry with an image analysis system of ImageQuant (Molecular Dynamics, CA, USA).

### Immunohistochemistry

The NUCB-2 expression in the frozen embedded tumor tissues was detected by anti-NUCB-2 antibody (Santa Cruz, TX, USA). Briefly, 5 µm thick cryo-sections were stained with anti-NUCB-2 antibody (1:100 dilution) (Santa Cruz, USA) overnight at 4℃ and then incubated with 1:1000 dilution of biotinylated secondary antibody, followed by treated with avidin-biotin peroxidase complex (DAKO, CPH, Denmark) according to the manufacturer’s instructions. Finally, the samples were reacted with 3,3’-Diaminobenzidine (Sigma, MO, USA) until brown colors were developed and counterstained with Harris modified hematoxylin.The human gastric cancer tissue was performed as the negative control.

### ELISA

Human NUCB2/nesfatin-1 ELISA kit (CUSABIO, Wuhan, China) was used for determining nesfatin-1 in serum samples guided by the manufacturer’s instruction. Firstly, 100 µL of standards or samples were added into each well and incubated for 2 h at 37 °C. After removing the liquid, 100 µL of biotin-antibody (1x) were added to each well and incubated for 1 h at 37 °C; then, 100 µL of HRP-avidin (1x) were aspirated to each well and incubated for 1 h at 37 °C; followed by repeating aspiration/wash for five times, 90 µL of TMB substrates were added to each well and incubated for 30 min at 37 °C in darkness; finally, 50 µL of stop solution were added and prepared for optical density determination within 5 min, using a microplate reader at the wavelength of 450 nm.

### MTT assay

CNE1-OE and CNE2-KD cells and their corresponding negative control cells were cultivated in RPIM-1640 medium containing 10% fetal calf serum and plated at 2 × 10^3^ cells per well in 96-well tissue culture plates for six days. At every 24 h, 20 µL of 5 mg/mL MTT (Sigma, MO, USA) were added into the wells and incubated for another 4 h, then the medium was removed and 250 µL of dimethylsulfoxide (DMSO) were added to each well for 10 min at room temperature. The absorbance of each well was read at 490 nm with an EL310 Microplate Autoreader supplied by BiTek Instruments (VT, USA). MTT assay was performed in triplicate.

### Plate clone formation assay

CNE1-OE and CNE2-KD cells and their corresponding negative controls in RPIM-1640 medium containing 10% fetal calf serum were plated at 1 × 10^3^ cells per well in 96-well tissue culture plates, and cultured for 10 days. When visible clones were shown, the cells were fixed with 5 mL of methanol for 15 min and then stained with the recrystallized violet staining solution (2 g/L) for 60 min. At last, the Image J 1.48v (National Institutes of Health, MD, USA) was performed to calculate the number and area of the clones.

#### EdU assay

The transfected CNE1 and CNE2 cells and the controls were seeded into 96-well plates at 5 × 10^3^ cells per well and the EdU labelling assay was conducted to evaluate the cell proliferation efficiency based on the manual from the supplier (Ribo Bio, Guangzhou, China). Fluorescence microscope (Clontech, CA, USA) was employed to photograph the images of the labeled cell. The EdU makeup rate wascalculated by the Image J 1.48v (National Institutes of Health, MD, USA).

### Statistics

Differences between two independent groups were analyzed by the student’s t-test; while one-way ANOVA test with Bonferroni’s multiple comparison was adopted for analyzing differences among three or more independent groups. *P* < 0.05 between groups was considered as the significant difference. All experimental calculations were carried out by the GraphPad Prism version 6.0 (GraphPad Software, CA, USA).

## Results

### NUCB-2 overexpression was determined in nasopharyngeal carcinoma tissues

**Fig. 1:**
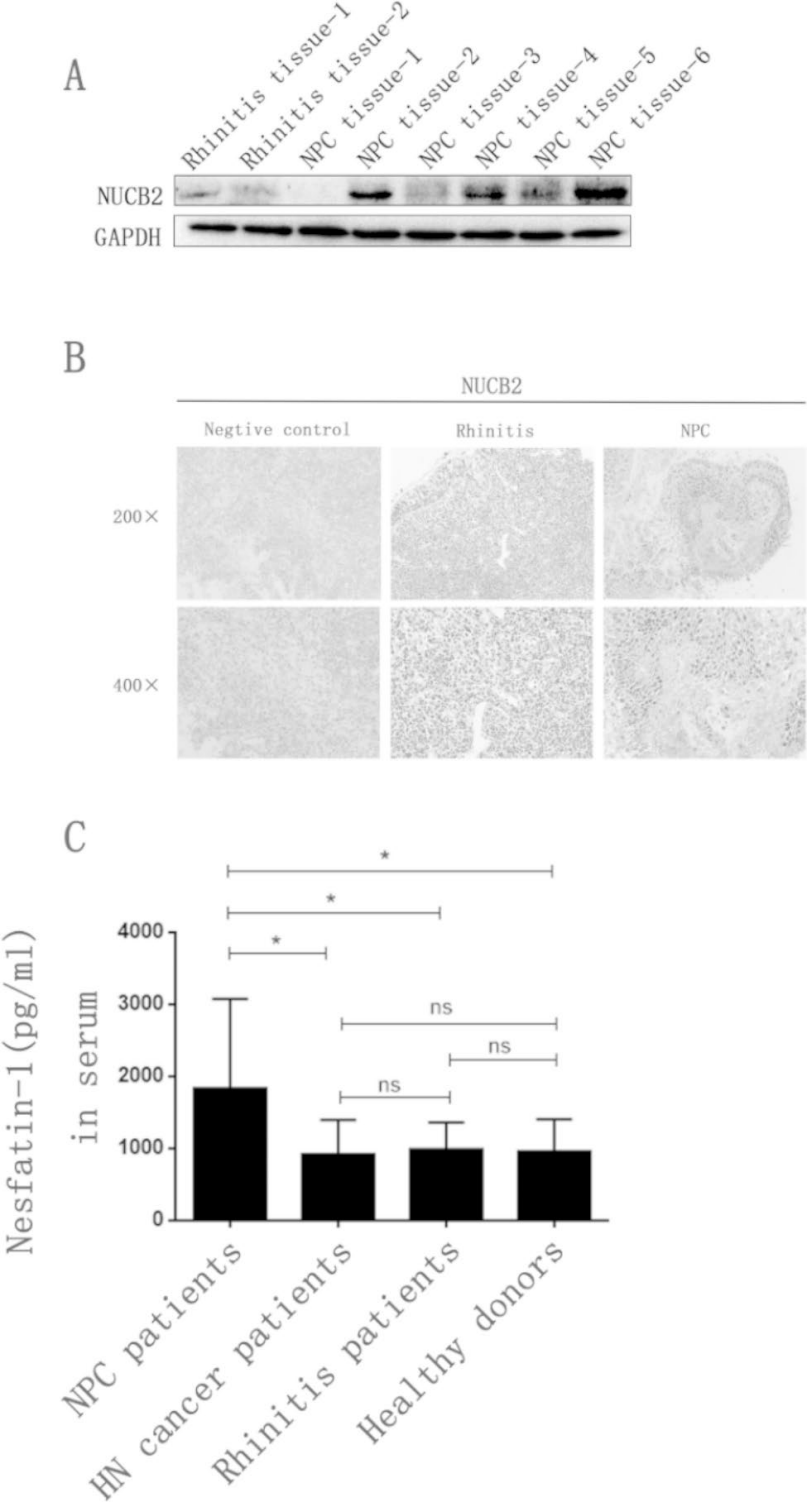
Comparison of of NUCB-2 expression between samples of clinical NPC and rhinitis. (**A**) Display of NUCB-2 expression detected by Western blot in six nasopharyngeal carcinoma tissues and two rhinitis controls, where GAPDH was used as the internal control of each sample. (**B**) Presentation of NUCB-2 expression detected by immunohistochemical staining on frozen sections of NPC and rhinitis, where human gastric cancer tissue served as the negative control. The NUCB-2 protein was stained brown in cytoplasm while the nuclei were stained blue. (**C**) Comparison of nesfatin-1 concentration in serum between patients with NPC (n = 49), head and neck (HN) cancer (n = 35), rhinitis (n = 20) and healthy donors (n = 36). *: *P* < 0.05; ns: non-significant with *P* > 0.05

To investigate the role of NUCB-2 in NPC, NUCB-2 expression was detected between 39 NPC samples and 31 rhinitis tissues. The Western blot and immunofluorescent staining results demonstrated that the level of NUCB-2 in tumor tissues was higher than that in rhinitis (Fig. 1A and B). To further determine whether NUCB-2 overexpression could result in systematic elevation, serum concentration of nesfatin-1, which is derived from the N-terminal of NUCB-2, was measured. The results revealed that the serum nesfatin-1 level in NPC patients significantly increased in comparison with that in head and neck tumor patients, rhinitis subjects or healthy donors (Fig. 1C). It suggests that NUCB-2/nesfatin-1 may not only locally but also systematically participate in the regulation of the signaling pathway in NPC cells.

### NUCB-2 overexpression was observed in poorly differentiated nasopharyngeal carcinoma cell line

**Fig. 2:**
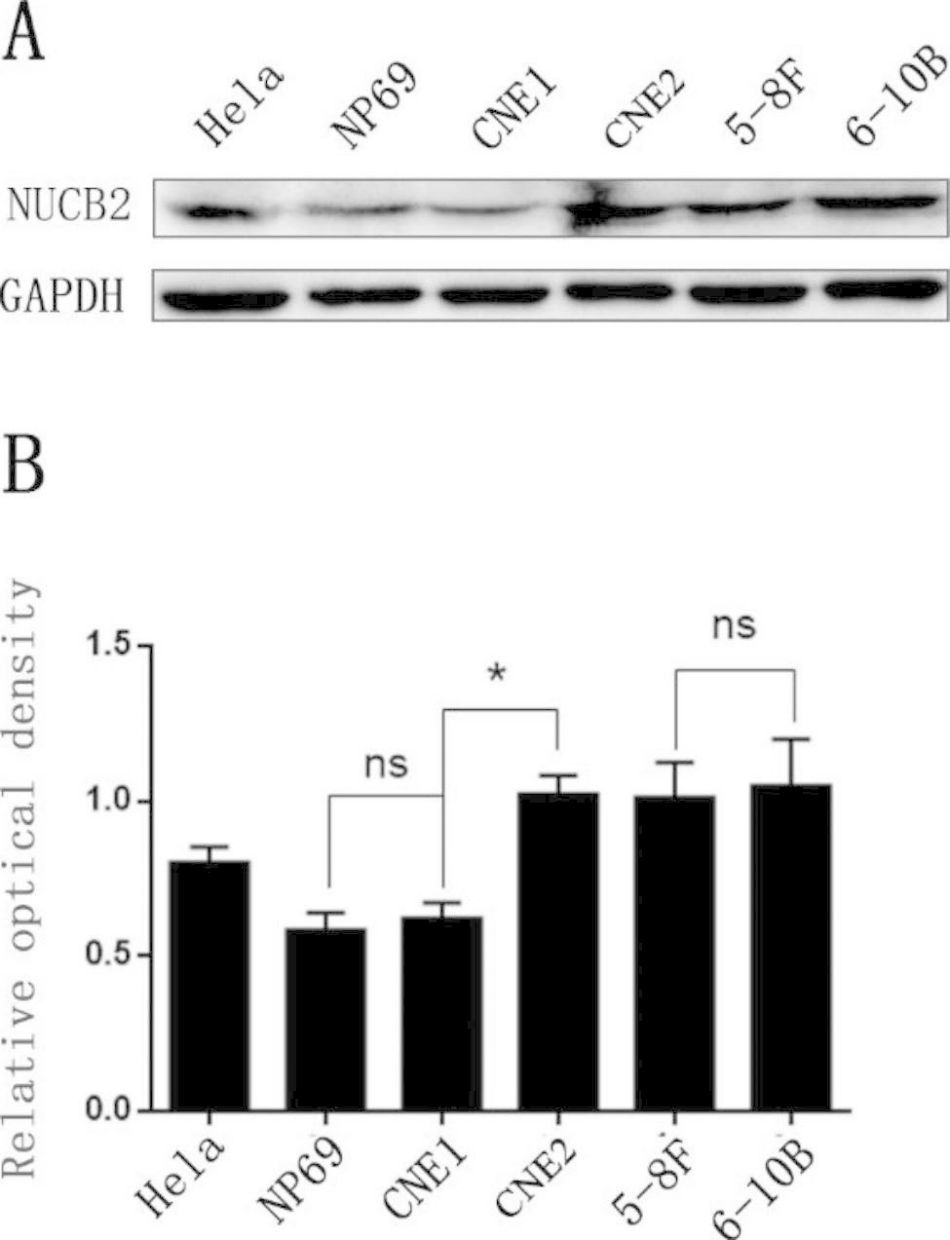
Comparison of NUCB-2 expression in different NPC cell lines (CNE1, CNE2, 5-8 F and 6-10B), while Hela cell was used as the positive control and NP69 as the control of normal immortalized nasopharyngeal mucosal cell. (**A**) Presentation of NUCB-2 expression determined by Western blotting in different NPC cell lines and the controls; GAPDH was detected as an internal control of each sample. (**B**) Comparison of NUCB-2 expression in different NPC cell lines and the controls. The relative optical density of NUCB-2 expression level was obtained as a ratio of NUCB-2 to the internal control of GAPDH in western blot analysis. *: *P* < 0.05; ns: non-significant with *P* > 0.05

The expression level of NUCB-2 was tested and compared between the immortalized nasopharyngeal mucosal epithelial cell line NP69, the highly metastatic NPC cell line 5-8 F, the low metastatic NPC cell line 6-10B, the highly differentiated NPC cell line CNE1 and the poorly differentiated NPC cell line CNE2, while Hela cells were used as the positive control [15]. The protein expression level of NUCB-2 in NP69 or CNE1 was significantly lower than that in CNE2 (*P* < 0.05), whereas there was no significant difference between NP69 and CNE1 (*P* > 0.05), nor between 5 and 8 F and 6-10B (*P* > 0.05) (Fig. 2A and B). These results imply that the high expression of NUCB-2 might promote the differentiation of NPC.

### Suppression of NUCB-2 inhibited the proliferation of the NPC CNE2 cell

**Fig. 3:**
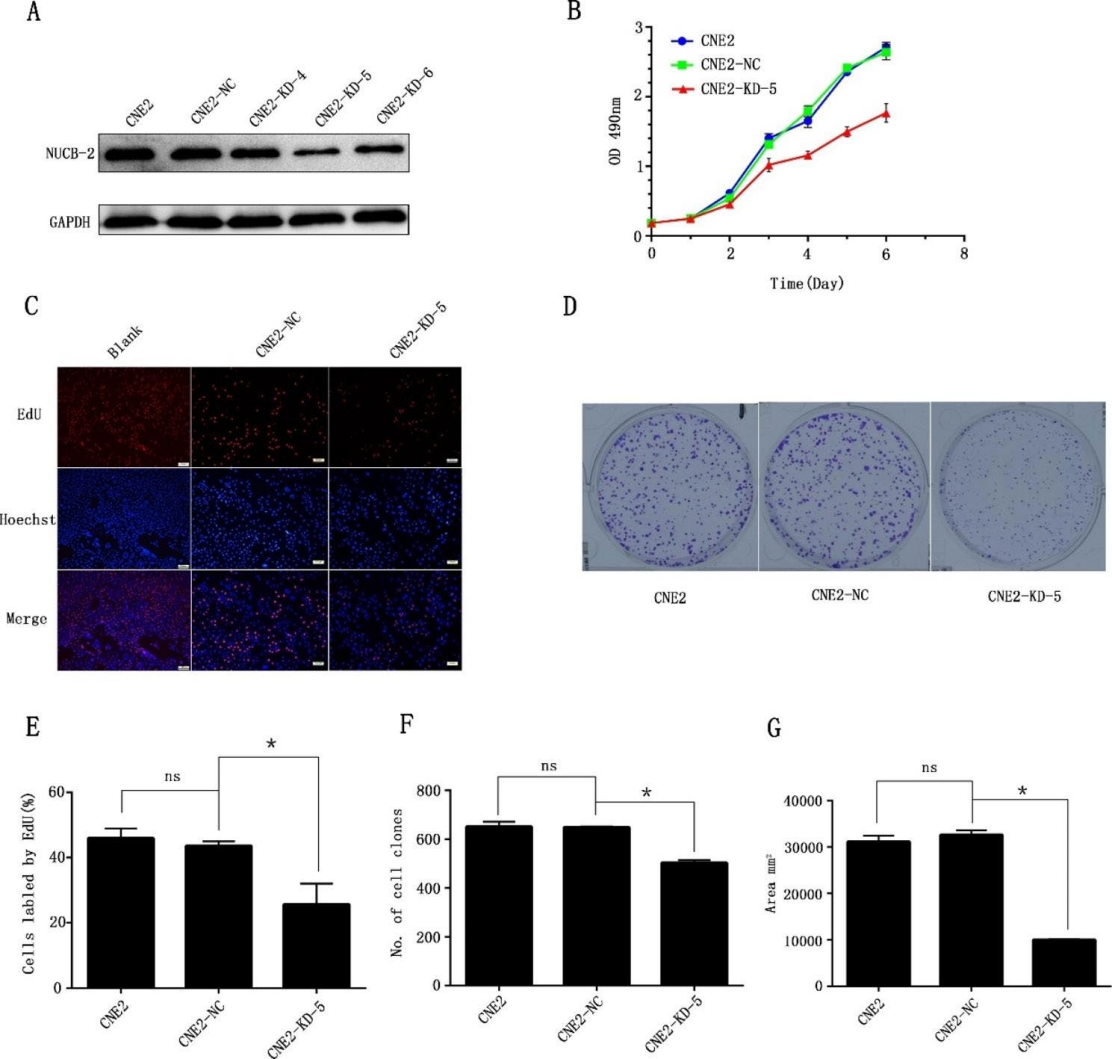
Suppressed NUCB-2 expression and its inhibition on CNE2 cell proliferation. (**A**) Western Blot detection on the effect of NUCB-2 expression in NUCB-2 knockdown clones of CNE2-KD-4, CNE2-KD-5 and CNE2-KD-6; GAPDH was employed as the internal control of each sample. (**B**) MTT assay. The x-axis represents the culture time of cells. The y-axis represents the absorbance of the cultured cells at the wavelength of 490 nm. (**C**) EdU assay. The figure displays the EdU fluorescence of CNE2-KD-5 cells. Blank: untreated CNE2 cells. (**D**) The results of the plate clone formation. (**E**) Histogram of the EdU results with the ImageJ software production, where the y-axis indicates the percentage of the labeled cells. (**F**) Histogram of the clone number of different CNE2 cell lines. (**G**) Histogram of the clone area (mm2). The ImageJ software was used for statistical analysis. Data are presented as the mean ± standard error. CNE2-NC: negative control, CNE2 cells transfected with the empty vector; *: *P* < 0.05; ns: no statistical difference

Since NUCB-2 was highly expressed in clinical NPC samples, to further investigate its function on NPC, NUCB-2 knockdown recombinants were established and transferred to the poor-differentiated CNE2 cells, namely, CNE2-KD. Western blot results showed that CNE2-KD-5 displayed greatly compromised expression of endogenous NUCB-2 protein, compared to other CNE2-KD cells (Fig. 3A), so it was selected for the follow-up experiments. MTT assay showed that the growth rate of CNE2-KD-5 decreased significantly (Fig. 3B). OD_490_ measurement and EdU assay revealed that CNE2-KD-5 cells exhibited slow proliferation after cultured for 48 h, in contrast to the controls (Fig. 3C and E). Moreover, the clone formation of CNE2-KD-5 was markedly blocked (Fig. 3D F and 3G). It indicates that suppression of NUCB-2 could slow down the growth of NPC cells.

### NUCB-2 overexpression enhanced proliferation of NPC CNE1 cell

**Fig. 4:**
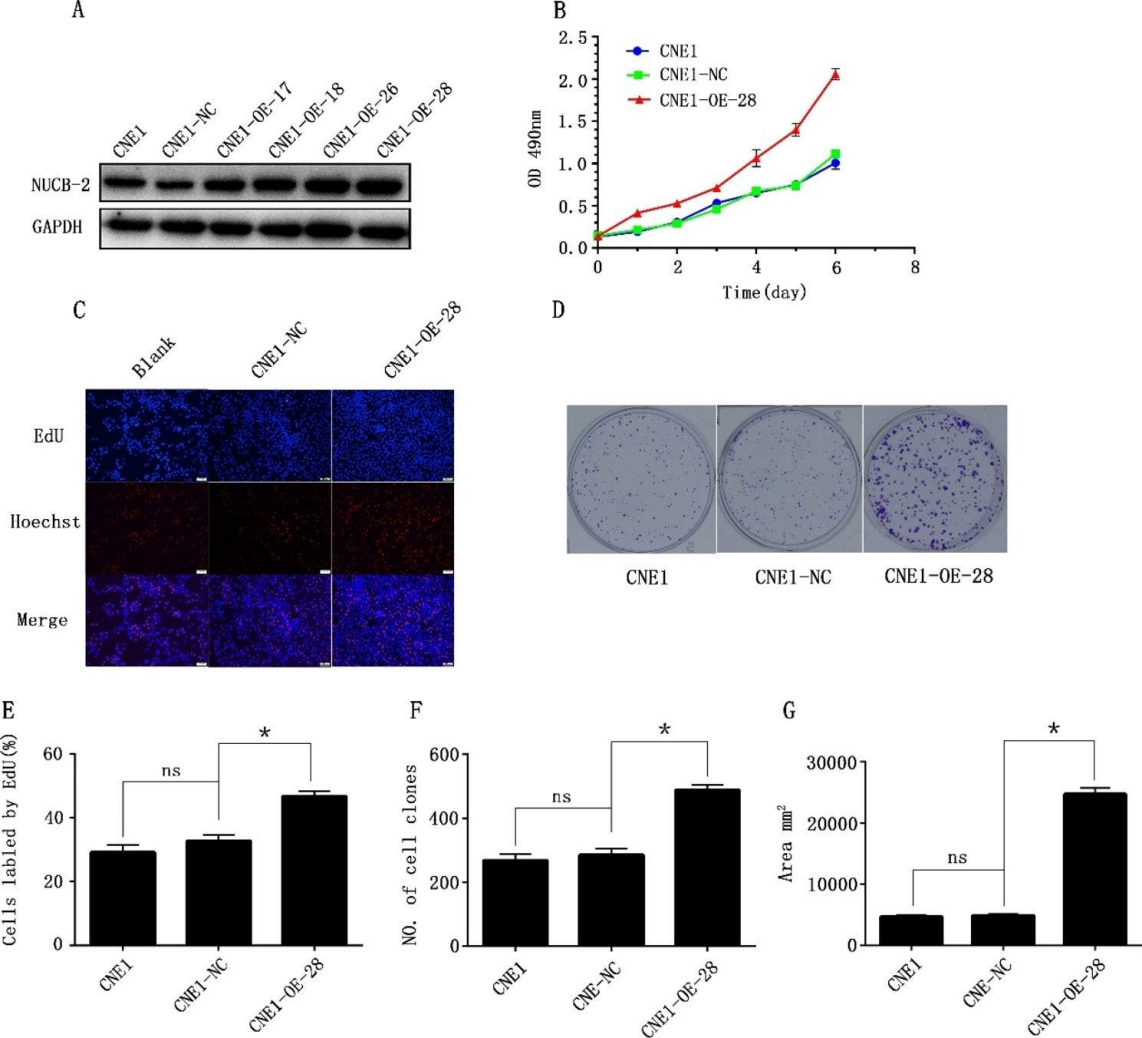
Overexpression of NUCB-2 and its promotion on CNE1 cell proliferation. (**A**) Western blot detection on the effect of NUCB-2 overexpression in different cell clones of CNE1-OE-17, CNE1-OE-18, CNE1-OE-26 and CNE1-OE-28; GAPDH: the internal control of each sample. (**B**) MTT assay. The x-axis represents the culture time of cells. The y-axis represents the absorbance of the cultured cell at the wavelength of 490 nm. (**C**) EdU assay. The figure shows the EdU fluorescence of NUCB-2 overexpression in CNE1-OE-28 cells; Blank: untreated CNE1 cells. The lower panel is the histogram of the EdU results with the ImageJ software production, where the y-axis indicates the percentage of labeled cells. (**D**) Plate clone formation assay. The image display the results of plate clone formation. (**E**) Histogram of the EdU results with the ImageJ software production, where the y-axis indicates the percentage of the labeled cells. (**F**) Histogram of the clone number of different CNE1 cell lines. (**G**) Histogram of the clone area (mm^2^).The ImageJ software was conducted for statistical analysis. Data are presented as the mean ± standard error. CNE1-NC: negative control, CNE1 cells transfected with the empty vector. *P < 0.05. ns: no statistical difference

To further determine the role of NUCB-2 on NPC cell proliferation, recombined expression vector containing NUCB-2 was constructed and transfected into the well-differentiated CNE1 cells, designated as CNE1-OE. Western blot detection demonstrated that NUCB-2 overexpression was seen in a number of stable CNE1-OE cell clones; among them, CNE1-OE-28 exhibited the strongest expression of NUCB-2, as shown in Fig. 4A, and it was selected for the next investigation. MTT assay showed that the growth rate of CNE1-OE-28 increased significantly (Fig. 4B). OD_490_ measurement and EdU assay manifested that CNE1-OE-28 cells exhibited significantly high proliferation efficiency (Fig. 4C) and EdU labeling rate (Fig. 4E) at 24 h post-culture. It suggests that over-expression of NUCB-2 promotes the proliferation of NPC cells. In addition, results of the plate clone formation assay proved that overexpression of NUCB-2 contributed to the colony formation of NPC cells in vitro (Fig. 4D F and 4G), implying the great importance of NUCB-2 for the growth and proliferation of the NPC cells.

### Clinical monitoring of NUCB-2/nesfatin level in the early diagnosis of NPC

**Fig. 5:**
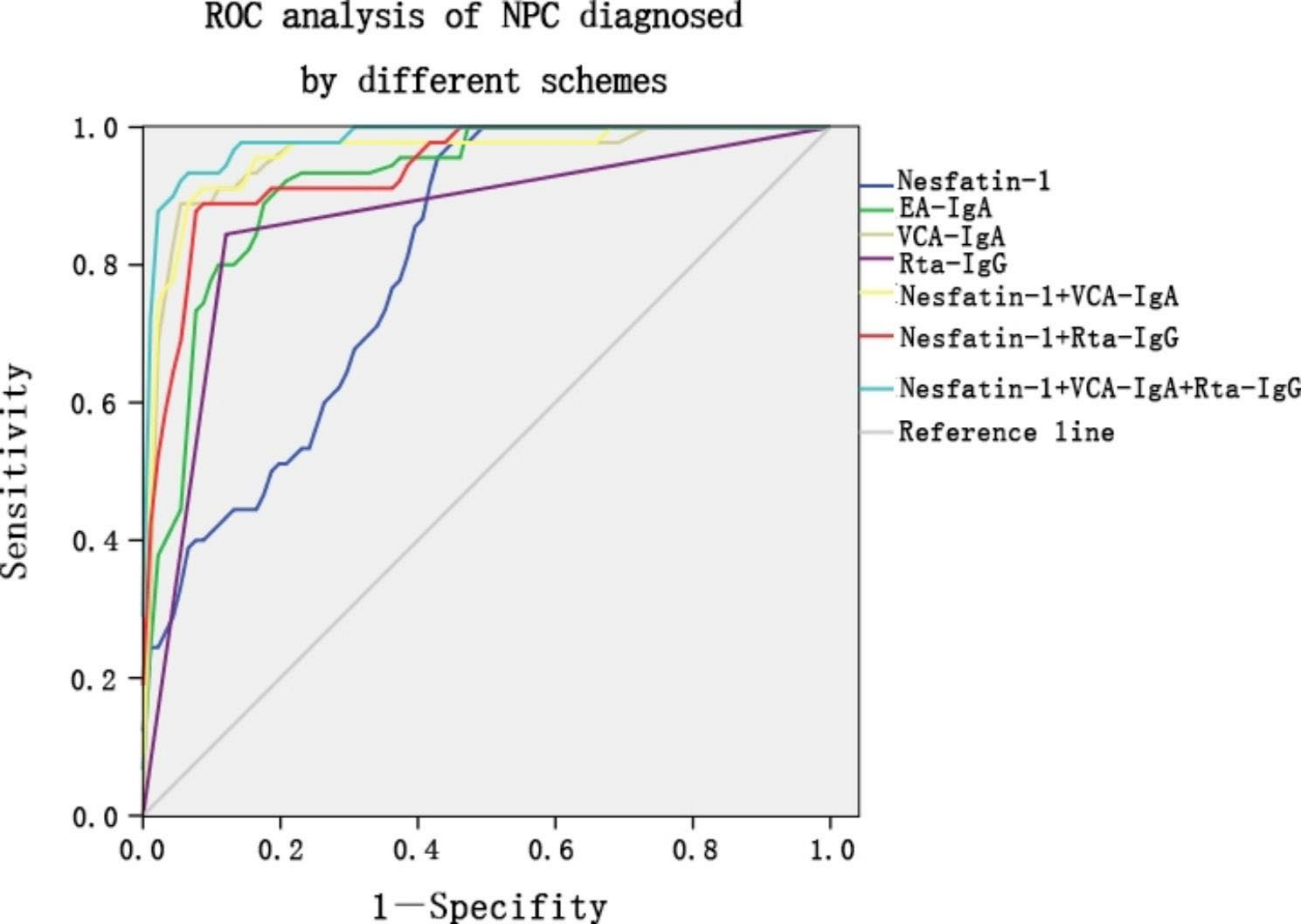
ROC curve analysis of nesfatin-1 and its combination with EB virus related antibody tests for NPC diagnosis. It displays that nesfatin-1 test combined with VCA-IgA and Rta-IgG for NPC diagnosis has the highest sensitivity and specificity compared to nesfatin-1 alone or its combination with other EB virus antibody tests

Our results show that NUCB-2 was important for NPC cell proliferation, we aimed to further explore whether NUCB-2 level could be used for early diagnosis of NPC. Firstly, expression of NUCB-2 was determined in the lesion sites of 39 patients initially diagnosed with NPC and 31 patients with rhinitis. Through chi square test, we found that the difference of NUCB-2 positive rate between the two groups was statistically significant (Table 1). The true positive rate of NPC detected by NUCB-2 immunohistochemistry was 56.4%, and the true negative rate was 67.7%. However, the correlation analysis revealed that the immunohistochemical score of NUCB-2 was not correlative to the Tumor-Node-Metastasis (TNM) stage of the tumor, suggesting that the immunohistochemical examination of NUCB-2 may be of great value for early diagnosis of NPC, especially for exclusion of the tumor. Our previous measurement showed that NUCB-2 overexpression resulted in systemic elevation of serum nespatin-1. Therefore, we further explored the clinical application value of serum nespatin-1 test coupled with EB virus antibody assays, including EA-IgA, VCA-IgA and Rta-IgG, in the diagnosis of NPC. In total, 140 blood specimens from Xiangya Hospital of Central South University, covering January 2017 to June 2017, were collected and nespatin-1 concentration in serum was assayed. The 140 cases were divided into four groups of primary NPC (n = 49), head and neck tumor (n = 35), rhinitis (n = 20) and healthy donors (n = 36). Among them, the primary NPC group displayed the highest nesfatin-1 level, which was statistically significant compared to the other three groups (*P* < 0.05), whereas serum nesfatin-1 levels between them were insignificant (*P* > 0.05). Moreover, nesfatin-1 had excellent sensitivity (97.9%) at diagnosing NPC, which would make up the shortfall of EA-IgA, VCA-IgA and Rta-IgG for their low sensitivity in NPC diagnosis. As described in Table 2, nesfatin-1 could improve its diagnostic efficiency when combined with VCA-IgA or Rta-IgG; noticeably, joint detection of the three indicators achieved the largest AUC^ROC^ (AUC = 0.982, 95%CI 0.963 ~ 1.000, *P* < 0.01). Nesfatin-1 test combined with VCA-IgA and Rta-IgG exhibits impressive sensitivity (93.6%) and specificity (94.5%) in the diagnosis of primary NPC, together with high positive and negative predictive values of 89.8% and 96.6%, while the accuracy reached 94.2% and yoden index was 0.881 (Fig. 5).

**Table 1:** Comparison of IH staining positive rate between NPC and rhinitis

Group	Positive (%)	Negative (%)	Chi-square value	DOF	Sig
NPC (n = 39)	56.4 (n = 22)	43.6 (n = 17)	4.06	1	0.044
Rhinitis (n = 31)	32.3 (n = 10)	67.7 (n = 21)			

IH: immunohistochemistry; DOF: degree of freedom; Sig: significance value

## Discussion

Identification of proteins related to NPC occurrence is important in discovering biomarkers for early warning NPC development. In this study, we firstly demonstrated that the expression of NUCB-2 in tumor regions was higher than that in non-tumor sites. On the other hand, a significant difference of serum nesfatin-1 level was observed between patients with NPC and head and neck cancer or rhinitis. Our results indicate that NUCB-2 overexpression in tumor may lead to the elevation of NUCB-2-derived-nesfatin-1 in serum and further tumor progression via systematical regulation of the relative signaling pathways. Therefore, we conclude that the expression of NUCB-2 in tumor tissue and nesfatin-1 in serum may have the potential to serve as risk factors for NPC progression.

NUCB-2 is distributed in multiple types of tissues, including adipose tissue, central neuron system, gastrointestinal system and reproductive organs, and has diverse functions in different tissues and cancers due to the distinct physiological features of each tissue and the involved various pathways [16–19]. Our studies provided evidences that NUCB-2 differentially expressed in NPC cell lines with different differentiation degrees, but not with different metastatic characteristics. Thus, we presume that NUCB-2 can enhance occurrence and development of NPC rather than promote metastasis or invasion of the tumor. Our MTT assay demonstrated that the multiplication rate of NUCB-2 knockdown NPC cells dramatically decreased at 48 h post-culture. The marking percentage of EdU, an excellent indicator of the DNA replication of the cells, was significantly low in NUCB-2 knockdown NPC cells in comparison with the controls. In addition, clone formation experiments proved that the number and size of the NUCB-2 knockdown NPC cells were significantly compromised in contrast to the controls. These in vitro experiment results suggest that following NUCB-2 suppression, DNA replication in NPC cells could be curbed, which ultimately cause suppressed proliferation of the tumor cells; conversely, with elevated NUCB-2 expression level, the proliferation and growth ability of the NPC cells were dramatically improved.

Although our experimental results strongly suggest that NUCB-2 contributes to NPC cell proliferation, the NUCB-2 signaling pathways are currently not well known. Some studies disclosed that the expression of NUCB-2 was regulated by multiple signaling pathways. In a NUCB-2-knockout renal cancer cell line SK-RC-52, the expression levels of molecules associated with epithelial-mesenchymal transition (EMT), including E-cadherin, β-catenin, Slug and Twist, were affected by NUCB-2 suppression [16]. Another investigation found that the AMP-dependent protein kinase (AMPK)/target of rapamycin complex (mTORC) 1 signaling pathway participated in the regulation of NUCB-2-mediated metastasis and EMT [17]. FTX played an oncogenic role in LUAD and contributed to cancer development via targeting miR-335-5p/NUCB-2 axis [18]. In colon cancer, NUCB-2/nesfatin-1 enhanced migration, invasion and EMT through LKB1/AMPK/TORC1/ZEB1 pathways in vitro and in vivo [19]. Over the last years, NUCB-2 has been discovered related to energy homeostasis, e.g. energy expenditure and glucose homeostasis, and the energy stress might be a key factor in controlling AMPK/TORC1 pathways in NUCB-2 knockdown colon cancer cells [20]. NUCB-2 mRNA level in the mouse’s oviduct was ascended after PMSG injection, but there was no significant change following hCG inoculation; nonetheless, NUCB-2 mRNA level was markedly reduced after ovariectomy, while recovered with 17 β-estradiol (E2) injection, but not by progesterone, which demonstrated that expression of NUCB-2/nesfatin-1 is regulated by E2 from the ovaries [21]. The above studies suggest the NUCB-2 links to multiple pathways in various tissues and affects their functions via interfering with the signaling. A latest research show that NUCB-2 might function as the versatile Ca^2+^ sensor involved in signal transduction [22]. To the best of our knowledge, only one report that did not include clinical sample validation initially explored that mir-30a-5p regulates the growth and metastasis of nasopharyngeal carcinoma by targeting NUCB2.

In the present study, the expression of NUCB-2 in NPC tissues associated with the pathological aggression traits of the tumor. NPC cells characterized by high proliferation appeared to express high level of NUCB-2 and relate to bad prognosis. This is also consistent with other relevant studies [23–25]. In accordance with the immunohistochemical expression of NUB-2, the nesfatin-1 level in serum in NPC patients was obviously increased. The increment of serological nesfatin-1 may be caused by continuously overexpressed NUCB-2 in NPC tissue; following lysis of the NPC cell, NUCB-2 is released from the cell and digested by prohormone convertase (PC) and caspase, then nesfatin-1, the main fragment of NUCB-2, is secreted into the blood, which consequently lead to its elevation in serum. Moreover, the results of ROC analysis suggest that NUCB-2 detection is of great significance for the early diagnosis of NPC; combination of serological nefastin-1 with EB virus antibodies is effective to improve the detection rate of early nasopharyngeal carcinoma. Unfortunately, we have not yet completed the study of the molecular mechanism of NUCB2 regulating NPC proliferation, which is the deficiency of this paper. However, the relevant experiments have been designed and are constantly advancing.

Despite the limitation of this study that, as it is a cross-sectional survey, the effects of NUCB-2 on the progression of NPC have not been explored adequately, to the best of our knowledge, this is the first study involving a large number of clinical samples to verify the regulation of NUCB2 on the occurrence and development of nasopharyngeal carcinoma. By determining expression level of NUCB-2/nesfatin-1 in NPC cell lines, clinical tumor tissues and serum samples, our findings expand the possibility of NUCB-2/nesfatin-1 as a predictive biomarker for NPC development, and may contribute to develop novel therapeutics against primary NPC by targeting NUCB-2/nesfatin-1 involved signaling pathways.

## Data Availability

All data generated or analysed during this study are included in this published article.
